# Genetic analysis of the X chromosome associates loci with progression of Parkinson’s disease

**DOI:** 10.1002/mds.30252

**Published:** 2025-06-03

**Authors:** Yu Liao, Hao Wu, Junhao Wang, Jean-Christophe Corvol, Jodi Maple-Grødem, Meghan C. Campbell, Alexis Elbaz, Alexis Brice, Michael A. Schwarzschild, Pille Taba, Sulev Kõks, Thomas G. Beach, Guido Alves, Ole-Bjørn Tysnes, Joel S. Perlmutter, Baijayanta Maiti, Jacobus J. van Hilten, Roger A. Barker, Caroline H. Williams-Gray, Clemens R. Scherzer, Ganqiang Liu

**Affiliations:** 1 Shenzhen Key Laboratory of Systems Medicine in Inflammatory Diseases, School of Medicine, Shenzhen Campus of Sun Yat-sen University, Shenzhen, Guangdong, 518107, China; 2 Department of Medical Informatics and Neurobiology Research Center, School of Medicine, Shenzhen Campus of Sun Yat-sen University, Shenzhen, Guangdong, 518107, China; 3 Sorbonne Université, Institut du Cerveau – Paris Brain Institute - ICM, Institut National de la Santé et de la Recherche Médicale, Centre National de la Recherche Scientifique, Assistance Publique Hôpitaux de Paris, Département de Neurologie et de Génétique, Hôpital Pitié-Salpêtrière, F-75013, Paris, France.; 4 The Centre for Movement Disorders, Centre for Brain Health, Stavanger University Hospital, 4011, Stavanger, Norway.; 5 Department of Chemistry, Bioscience and Environmental Engineering, University of Stavanger, 4021, Stavanger, Norway.; 6 Departments of Neurology and Radiology, Washington University School of Medicine, St. Louis, MO 63110, USA.; 7 Université Paris-Saclay, UVSQ, Inserm, Gustave Roussy, CESP, 94805, Villejuif, France.; 8 Department of Neurology, Massachusetts General Hospital and Harvard Medical School, Boston, MA 02114, USA.; 9 Department of Neurology and Neurosurgery, Institute of Clinical Medicine, University of Tartu, Tartu, 50406, Estonia.; 10 Neurology Clinic, Tartu University Hospital, Tartu, 50406, Estonia.; 11 Centre for Molecular Medicine and Innovative Therapeutics, Murdoch University, Murdoch, Perth, 6150 WA, Australia.; 12 Perron Institute for Neurological and Translational Science, Nedlands, WA 6009, Australia.; 13 Banner Sun Health Research Institute, Sun City, AZ, USA.; 14 Department of Neurology, Stavanger University Hospital, 4068, Stavanger, Norway.; 15 Department of Neurology, Haukeland University Hospital, 5020, Bergen, Norway.; 16 Department of Clinical Medicine, University of Bergen, 5020, Norway.; 17 Departments of Radiology and Neuroscience, Washington University School of Medicine, St. Louis, MO 63110, USA.; 18 Program of Physical Therapy and Program of Occupational Therapy, Washington University School of Medicine, St. Louis, MO 63110, USA.; 19 Department of Neurology, Leiden University Medical Center, Albinusdreef 2, 2333 ZA, Leiden, The Netherlands.; 20 John Van Geest Centre for Brain Repair, Department of Clinical Neurosciences, University of Cambridge, Cambridge, CB2 0PY, UK.; 21 Wellcome - MRC Cambridge Stem Cell Institute, University of Cambridge, Cambridge, CB2 0AW, UK.; 22 Stephen & Denise Adams Center for Parkinson’s Disease Research of Yale School of Medicine, New Haven, CT 06510, USA; 23 APDA Center for Parkinson Precision Medicine, Yale, New Haven, CT 06510, USA; 24 Department of Neurology, Yale, New Haven, CT 06510, USA; 25 Department of Genetics, Yale, New Haven, CT 06510, USA; 26 Guangdong Provincial Key Laboratory of Brain Function and Disease, Guangzhou, 510080, China

**Keywords:** Parkinson’s disease, progression, X-chromosome-wide survival study, eQTL

## Abstract

**Background:**

Genetic variants on the X chromosome have been linked to susceptibility for Parkinson’s disease (PD), but their roles in disease progression remain unclear.

**Objectives:**

This study investigated associations between X chromosome variants and longitudinal cognitive decline or motor impairment in patients with PD.

**Methods:**

We conducted combined (male + female) and stratified X-chromosome-wide survival studies (XWSS) in 4,467 PD patients with 33,406 longitudinal visits. Cognitive decline was defined as global cognitive impairment (GCI, Mini Mental State Exam score ≤ 25), while motor impairment was evaluated by Hoehn and Yahr stage 3 (HY3). Expression quantitative trait locus (eQTL) and genetic colocalization analyses were systematically performed.

**Results:**

We identified 40 common variants in the X-chromosome-wide screen associated with longitudinal progression of PD with *P*-value < 9.27 × 10^−6^, including 11 independent loci associated with cognitive decline and two with motor impairment. The rs142724191 and rs144112368 variants were associated with cognitive decline in both combined and male-only analyses. rs111708875 reached genome-wide significance for motor progression in female cases (hazard ratio [HR] = 3.98, 95% confidence interval [CI] = 2.54–6.25) with *P*-value = 1.84×10^−9^. All these variants were independent with X chromosome susceptibility loci associated with PD, Alzheimer’s disease (AD), or Lewy body dementia (LBD).

**Conclusions:**

Our XWSS identifies novel genetic progression-associated loci on the X chromosome for PD, providing new insights into the X chromosome-linked genetic underpinnings of Parkinson’s disease.

## Graphical Abstract

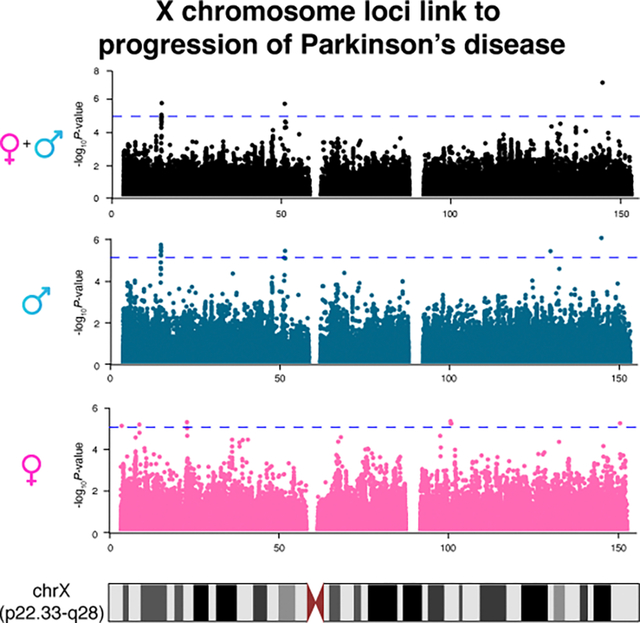

X-chromosome-wide survival study (*N* = 4,467 PD patients) identified 40 progression-associated variants (*P* < 9.27 × 10^−6^), including 11 loci linked to global cognitive impairment and two loci linked to motor impairment. These variants are independent of PD, AD, or LBD susceptibility loci, revealing novel X chromosomal genetic underpinnings of PD progression.

## Introduction

Parkinson’s disease (PD), the second most common neurodegenerative disorder^1^, currently affects millions worldwide, imposing substantial burdens on patients, their families, and healthcare systems^2^. Epidemiological data reveal males have a 1.5-fold higher risk of developing PD than females^3^, while a recent study reported an updated overall male-to-female prevalence ratio (OPR) of 1.18 (95% confidence interval [CI]: 1.03–1.36)^4^. In motor symptoms, female patients may manifest a comparatively less severe phenotype, characterized by increased tremor and less pronounced motor impairment, while also exhibiting a higher susceptibility to developing dyskinesias than male patients with PD^5, 6^. In cognitive decline, male patients emerge as a crucial determinant of early cognitive decline, whereas females typically experience a slower trajectory towards cognitive impairment^7^. Furthermore, a few neuroimaging studies have explored the differences between males and females in PD. Males displayed notable reductions in cortical thickness, compared with those in female PD patients^8^. Notably, male patients had significantly greater tissue loss than females in 11 cortical regions including bilateral frontal and left insular lobe, while females had greater atrophy in six cortical regions^9^.

Accumulating evidence has shown the integral role of genetics in both the onset^10^ and progression of PD^11–15^. Large-scale multi-ancestry genome-wide association studies (GWAS) have reported several potentially novel loci of PD^16^. Nevertheless, these variants elucidated only about 16–36% of the heritability in sporadic PD. Familial cases accounted for approximately 15% of PD cases^17^, with more than 20 gene mutations related to familial PD have been identified^18^, including *SNCA*^19, 20^, *PRKN*^21^ and *LRRK2*^22^. A genome-wide survival study (GWSS) has identified novel genetic risk loci specific to PD dementia (PDD)^11^.

Most of these studies were concentrated on autosomes. The X chromosome, which constitutes 5% of the human genome and spans approximately 155 Mb, has been underrepresented in most GWAS due to its unique features such as male hemizygosity and different quality control requirements compared to autosomes^23^. Khramtsova and colleagues established standardized pipelines for X chromosome analysis, including quality control procedures, imputation methods, and models (XWAS)^24^. Recent case-control studies leveraging X chromosome data from PD cohorts identified genetic variants linked to PD risk^25, 26^. However, the relationship between X chromosome variants and the longitudinal PD progression remains unexplored.

In this study, we employed an X-chromosome-wide survival study (XWSS) to investigate the impact of variants on the X chromosome on longitudinal cognitive or motor progression in 4,467 PD patients with multiple longitudinal cohorts. We performed both combined and stratified analyses to identify X-chromosome genetic variants associated with PD progression and to discern potential influences. We identified several novel variants on the X chromosome linked to global cognitive impairment (GCI) or motor progression (HY3). Further exploratory analysis identified *cis*-expression quantitative trait (*cis*-eQTL) loci linked to these variants.

## Materials and methods

### Study participants

This study analyzed data from 4,896 PD patients (24 without clinical visits) across 15 cohorts in North America and Europe, encompassing 36,123 clinical follow-up visits spanning 1986–2017. Of these patients, 4,384 were genotyped using the Illumina Multi-Ethnic Genotyping Array (MEGA), and 512 underwent whole genome sequencing (WGS). Detailed descriptions of patient information across 15 cohorts have been thoroughly provided in the study by Liu et al^11^. Written informed consent for DNA collection and phenotypic data collection for secondary research use for each cohort was obtained from the participants with approval from the local ethics committees. The Institutional Review Board of the School of Medicine, Sun Yat-sen University approved the current analyses.

### Data quality control and imputation

The MEGA genotyping data was gathered by the International Genetics of Parkinson’s Disease Progression (IGPP) Consortium^11^. Prior to X-chromosome quality control (QC), 4,384 PD patients using MEGA (24 without clinical records) and 512 PD patients using WGS, with 1,779,819 SNPs underwent QC, resulting in 4,491 PD patients (4,020 with MEGA genotyping data and 471 with WGS data) and 1,635,580 SNPs passing QC. The details of prior to X-chromosome QC have been reported in Liu et al^11^. In this study, the details of X chromosome genotyping data quality control and imputation were shown in Supplementary Fig. 1. Male or female was determined using genetic data and validated through a check analysis conducted in PLINK (version 1.9)^27^. All patients were confirmed to be consistent with self-reported questionnaires.

X chromosome genotyping data QC and imputation followed a five-step pipeline: *pre-imputation quality control*, *imputation*, *ChrX post-imputation quality control*, *merge PPMI PD patients*, and *final ChrX quality control*. The complete QC pipeline was described in Supplementary Methods. After QC, a total of 4,467 PD patients (3,996 MEGA and 471 WGS), including 2,822 males and 1,645 females across 15 cohorts (Supplementary Table 1), encompassing 33,406 clinical follow-up visits, with 213,758 X chromosome SNPs were retained for downstream analysis.

### Statistical analysis

For 4,467 PD patients, the median number of follow-up visits per participant was 6 (interquartile range [IQR] = (3, 11)), with a median interval between consecutive follow-ups of 0.47 years (IQR = (0.24 years, 0.89 years)). 31,885 (95.4%) of visits occurred within 12 years of longitudinal follow-up from disease onset with a median follow-up time of 6.70 years (IQR = (4.59 years, 8.81 years)). We therefore focused our survival analyses on the 12-year time frame from disease onset. We utilized the Cox proportional hazards (CoxPH) analysis in R (version 3.6.3) to estimate the influence of risk factors on the time (years from PD disease onset) to reach the endpoint. The ‘survival’ package (version 3.5.5) was employed to investigate the impact of each X chromosome genetic variant (213,758 SNPs passed QC) on the time to reach GCI as indicated by a Serial Mini Mental State Exam (MMSE) score ≤ 25, which was in line with the recommendations of the International Parkinson and Movement Disorder Society (MDS) Task Force^28^. Here, we adjusted for several covariates: sex (in the combined analysis only), age at onset (defined as the patient’s age at the time of PD diagnosis for most studies; PROPARK and PROPARK-C defined age at onset as the patient’s age at the time of first patient-reported motor symptoms, as reported by Liu et al^12, 13^), years of education, the first 10 principal components (PCs) calculated from autosomes, and the polygenic hazard score as per Liu et al^11^. We also incorporated a cohort term as a random effect, employing a frailty model^29^. Additionally, we explored a second endpoint: motor disability with postural instability, as defined by Hoehn and Yahr stage = 3 (HY3)^30^. This analysis was adjusted for sex (in the combined analysis only), the first 10 PCs from autosomes, and age at onset, with the study name serving as a random term. For cognitive impairment analysis, 704 patients including 299 left-censored and 405 with missing or non-quality clinical data were excluded. Similarly, 732 patients (439 left-censored) were excluded for motor impairment analysis. Right-censored patients were deemed not to have met the study endpoint criteria and were therefore maintained in the analytical cohort.

To address cohort-specific consistency, we analyzed the two largest cohorts, HBS (*N* = 573, 65.3% male) and PPMI (*N* = 471, 61.6% male) using CoxPH analysis. Here, we excluded the “cohort” random effect, and kept all other parameters in the XWSS analysis. Only CoxPH models with more than 10 events (defined as occurrences of GCI or HY3) were reported (Supplementary Table 2).

The linkage disequilibrium score regression (LDSC) intercept was calculated with an ancestry-matched LD reference panel from the 1000 Genomes EUR.v3 (https://www.internationalgenome.org/) for each XWSS analysis within the LDSC (v1.0.1)^31^. When the LDSC intercept exceeded 1.05, we corrected test statistics for each SNP based on the LDSC intercept. Specifically, the χ^2^ association statistics for each SNP were scaled by dividing the LDSC intercept. Then the adjusted *P*-values for each SNP were recalculated using a chi-squared distribution with one degree of freedom based on scaled statistics.

Our study included a combined analysis encompassing all PD patients, alongside stratified analyses for males and females separately. For the male subset, we modeled genotypes as 0/2, consistent with the standard approach of considering males equivalent to female homozygotes^24^.

The number of haplotype blocks was estimated using the linkage disequilibrium method, in accordance with Haploview’s definition of haploblock^32^. The ‘blocks-max-kb 1000’ parameter in PLINK was applied to estimate the total number of haplotype blocks on the X chromosome for 4,467 PD patients with 213,758 SNPs that passed QC. Similar to the approach in Bayram et al.^33^, the Bonferroni threshold for X-chromosome-wide significance was 9.27 × 10^−6^ (0.05/5,395 haplotype blocks) in our study. The genome-wide significance threshold was set at *P*-value < 5 × 10^−8^.

The LocusZoom (version 1.4)^34^ was employed to investigate the association strength, local LD, recombination rate and gene positional information of significant SNP markers within a specific genomic region using Python (version 2.7.17).

To explore whether genetic variants identified in our XWSS are novel risk loci for the progression of PD, we conducted a comparative analysis with known genetic markers on the X chromosome linked to PD^25, 26^, Alzheimer’s disease (AD)^35^, or Lewy body dementia (LBD)^33^. We downloaded EUR.v3 X chromosome genomic data (total 503 samples) from the 1000 Genomes Project and used PLINK to select SNPs that showed significant linkage disequilibrium (r^2^ > 0.6) with the independent SNPs in GCI and HY3 (85 SNPs in GCI, 49 SNPs in HY3). These SNPs were then compared to those showing significant linkage disequilibrium (r^2^ > 0.6) with susceptibility SNPs for AD, PD, or LBD (total 485 SNPs showed LD with 11 susceptibility SNPs in Supplementary Table 3), ensuring a focused examination of potential unique genetic signatures and overlaps.

### Function annotation

We used the Functional Mapping and Annotation of Genome Wide Association Studies (FUMA; version 1.6.0)^36^ tool, an accessible online resource (http://fuma.ctglab.nl), for selecting independent SNPs. The following parameters were used in this analysis: maximum *P*-value of lead SNPs < 9.27 × 10^−6^, maximum *P*-value cutoff < 0.05, r^2^ threshold ≥ 0.6 to define independent SNPs and r^2^ threshold ≥ 0.1 to define lead SNPs, referencing the 1000 Genomes Phase3 EUR as the population panel and a maximum distance of < 250 kb between LD blocks for locus merging.

### Genetic colocalization analysis

An eQTL is a genetic variant linked to changes in the expression level of a gene. We employed X chromosome eQTL data from various sources, including Genotype-Tissue Expression Project^37^ (GTEx, version 8, https://gtexportal.org/), where eQTLs with False Discovery Rate (FDR)-corrected *P*-values < 0.05 were available, and others from brain tissue (CommonMind^38^, Braineac2^39^), monocytes (CEDAR^40^), microglia (Young et al., 2019)^41^ and T cells (Kasela et al., 2017)^42^, where eQTLs with raw *P*-values were used for our analysis. All eQTL data were uniformly processed via the eQTL catalogue^43^.

Colocalization describes a statistical approach to determine if two traits (e.g., a disease and altered gene expression) are influenced by the same causal genetic variant. Detailed methods, including statistical parameters and tools used for colocalization, are provided in the Supplementary Methods.

## Result

### Association between X chromosome variants and cognitive decline during the course of PD

**Figure 1:**
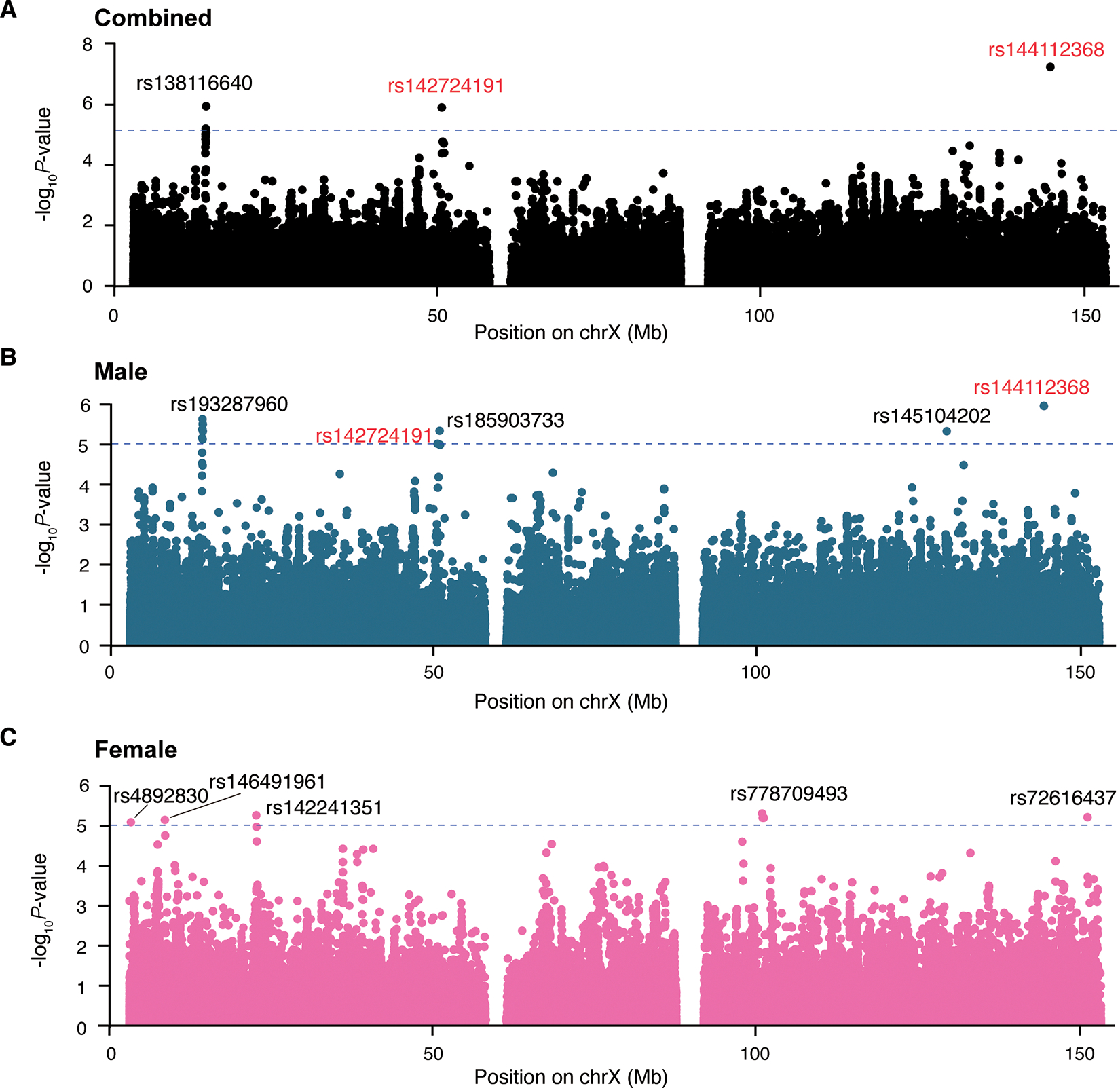
Manhattan plots show associations between common X-chromosome variants and risk of global cognitive impairment during disease progression. We conducted combined (**A**), male-only (**B**), and female-only (**C**) analyses. The dashed blue horizontal line indicates the X-chromosome-wide significance threshold (*P*-value < 9.27 × 10^−6^). The x-axis represents the positions of SNPs (hg19), while the y-axis represents the −log_10_(*P*-value) in the XWSS using Cox proportional hazards models with a two-sided Wald test. The marked red rs142724191 and rs144112368 were identified in both combined and male-only analyses.

To estimate the association between genetic variants and time (years from onset of PD) to achieving the GCI endpoint during the progression of PD, we analyzed data from 3,763 PD cases, of which 12.7% (478/3,763) reached the endpoint. The cohort comprised 2,370 males and 1,393 females, with 30,844 longitudinal study visits. In the combined analysis, we identified 11 significant SNPs with X-chromosome-wide associations with GCI (*P*-value < 9.27×10^−6^), 27 significant SNPs in male-only analysis and seven SNPs in female-only analysis (a total of 34 significant unique SNPs from combined and stratified analysis, Fig. 1). The quantile–quantile (QQ) plot of the XWSS analysis revealed inflation (genomic inflation factor (λ_GC_) = 1.055–1.135) (Supplementary Fig. 2A–C), yet the LDSC intercept estimates (0.977–1.003) demonstrated that this inflation primarily reflected polygenic architecture rather than confounded by population stratification. It should be noted that the signals of rs142724191 and rs144112368, located near *RP11–104D21.3* and *MIR513C*, respectively, passed the X-chromosome-wide association threshold in both combined and male-only analysis. In the combined analysis, rs142724191 demonstrated a hazard ratio (HR) of 1.73 (95% CI: 1.38–2.15; *P*-value = 1.26×10^−6^), with a consistent effect observed in males (HR = 1.72, 95% CI: 1.35–2.18; *P*-value = 9.01×10^−6^). Similarly, rs144112368 showed stronger association signals, with an HR of 2.18 (95% CI: 1.64–2.88; *P*-value = 5.71×10^−8^) in the combined analysis. This association remained robust in males (HR = 2.18, 95% CI: 1.60–2.99; *P*-value = 1.02×10^−6^) (Table 1).

We further processed the 34 unique SNPs (*P*-value < 9.27×10^−6^) associated with cognitive decline into the FUMA pipeline, and identified 11 independent unique SNPs (Table 1). Among these, we identified three independent SNPs in the combined analysis, and five independent SNPs in the male-only and female-only analyses, respectively (Fig. 1). rs142724191 and rs144112368 variants were independent in both combined and male-only analyses. SNPs rs138116640, located near *AC003035.1* in combined analysis, and rs193287960, located near *GEMIN8* in male-only analysis, showed notable LD with multiple neighboring SNPs. This LD pattern was identified through localized analysis using LocusZoom. The LD patterns analysis revealed that rs138116640 was in linkage disequilibrium with 31 SNPs in the combined analysis (Supplementary Fig. 3A). Compared to non-carriers, rs138116640 carriers exhibited a significantly higher risk, with a HR of 3.23 (95% CI = 1.94 – 5.37, *P*-value = 6.36×10^−6^) for progression to GCI (Supplementary Fig. 4A). Additionally, rs193287960 was in linkage with 34 SNPs in the male-only analysis (Supplementary Fig. 3B). rs193287960 carriers exhibited a higher risk with a HR of 4.02 (95% CI = 2.26 – 7.14) and *P*-value = 2.18×10^−6^ (Supplementary Fig. 4B). We found that the independent SNPs rs138116640 and rs193287960 are in close genomic proximity and exhibit strong LD (r^2^ > 0.6), which may influence the same locus. LocusZoom analysis indicated no obvious LD with the other nine independent SNPs, which were located in different loci. Carriers of these independent SNPs also showed a significantly higher risk for GCI compared to non-carriers (Supplementary Fig. 4C–M).

The CoxPH analysis of individual cohort (HBS and PPMI) revealed partial consistency for 11 independent SNPs. In the HBS cohort, rs142724191 was associated with GCI in male-only analysis (*P*-value < 0.05). In the PPMI cohort, rs144112368 was associated with GCI in both combined and male-only analyses, while rs185903733 was associated in male-only analysis (*P*-value < 0.05) (Supplementary Table 2).

Based on 11 previously reported independent SNPs associated with the risk of developing PD (three SNPs)^25, 26^, AD (seven SNPs)^35^, or LBD (one SNP)^33^ on the X chromosome (Supplementary Table 3), we identified a total of 485 SNPs (134 SNPs in PD; 350 SNPs in AD; one SNP in LBD) in high linkage disequilibrium (LD) (r^2^ > 0.6) with these 11 SNPs. In our study, we identified 11 independent SNPs associated with PD-GCI and found 85 SNPs with high LD (r^2^ > 0.6). Notably, none of the 85 SNPs associated with GCI overlapped with the 485 SNPs linked to PD, AD, or LBD.

### Association between X chromosome variants and motor progression during the course of PD

**Figure 2:**
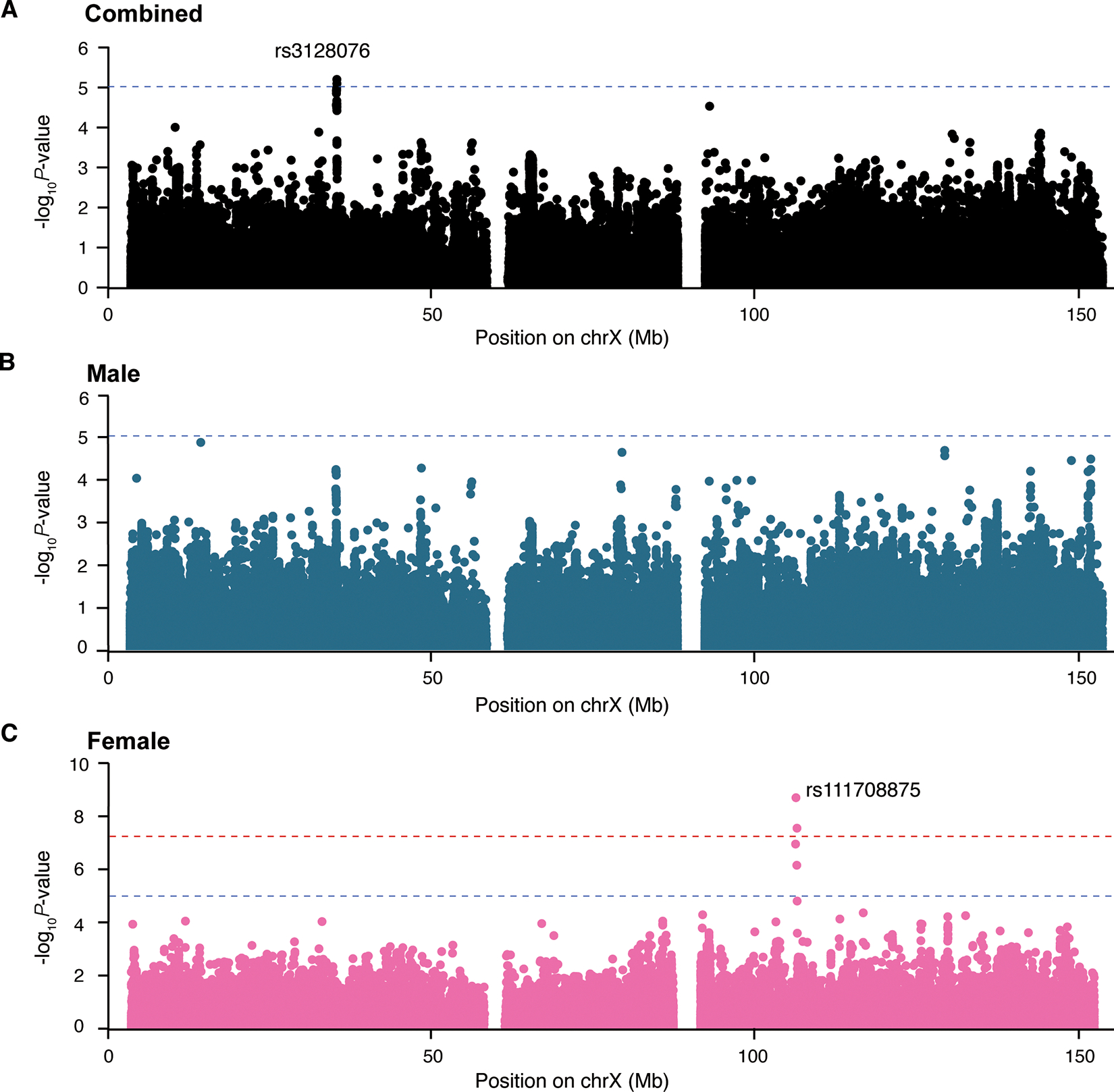
Manhattan plots of common X-chromosome variants associated with motor progression during PD. We conducted combined (**A**), male-only (**B**), and female-only (**C**) analyses. The dashed blue line corresponds to the X-chromosome-wide significance threshold (*P*-value < 9.27 × 10^−6^); The dashed red line corresponds to the genome-wide significance threshold (*P*-value < 5 × 10^−8^). The x-axis represents the positions of SNPs (hg19), while the y-axis represents the −log10(*P*-value) calculated by XWSS from the Cox proportional hazards model with a two-sided Wald test after correcting based on the LDSC intercept.

To explore if any SNPs on the X chromosome were linked to the motor impairment during PD progression, we performed XWSS analysis using motor disability with postural instability (Hoehn and Yahr stage = 3) as an endpoint in the longitudinal study. A total of 3,735 patients were included in this analysis (1,331 females and 2,404 males), with 23.9% (892/3,735) achieved the endpoint across the 30,959 longitudinal study visits. To address the moderate genomic inflation (λ_GC_ = 1.106–1.17) identified in the XWSS HY3 analysis (Supplementary Fig. 5A, C, E), where the LDSC intercept (1.089–1.111) indicated potential confounding biases, we corrected the test statistics based on the LDSC intercept. This adjustment successfully reduced genomic inflation, with post-correction λ_GC_ values ranging from 1.009 to 1.075 (Supplementary Fig. 5B, D, F). We identified two SNPs associated with motor impairment in the combined analysis and four SNPs in female-only analysis with *P*-value < 9.27×10^−6^ (total six significant unique SNPs, Fig. 2). Notably, rs111708875 (HR = 3.98, 95% CI = 2.54–6.25), located near *COL4A5,* reached genome-wide significance in the female-stratified analysis with *P*-value of 1.84×10^−9^ (Fig. 2).

**Figure 3:**
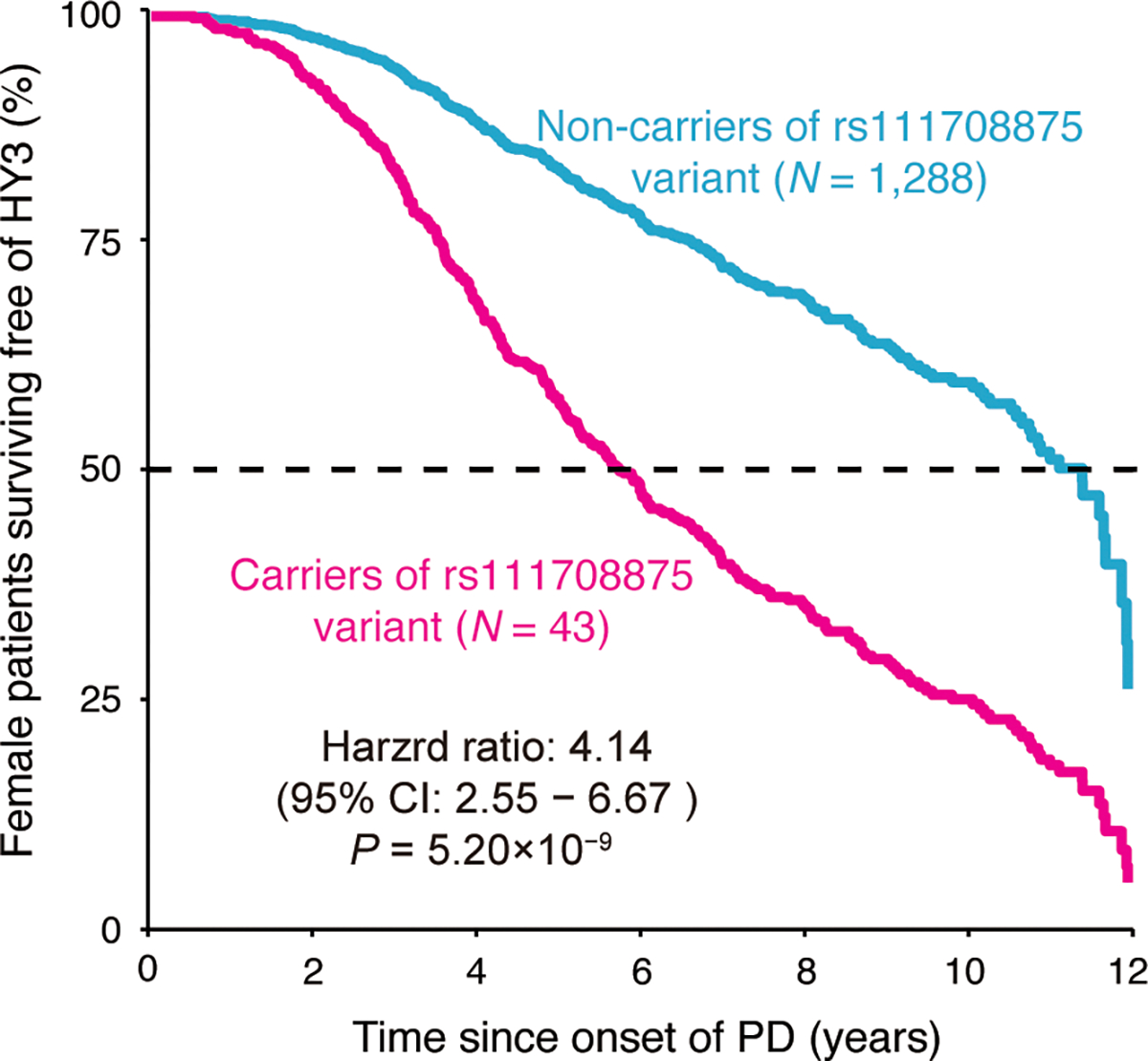
The rs111708875 accelerates motor progression in female-only PD patients. Covariate-adjusted survival curves for female patients with PD without rs111708875 (light blue line) and for those carrying the variant (magenta line) in the female-only analysis. The x-axis represents the years since the onset of PD, while the y-axis represents the percentage of female patients surviving free of motor impairment (HY = 3). Cox proportional hazards model with a two-sided Wald test.

For the six significant X chromosome SNPs associated with motor progression, we conducted a similar analysis using the FUMA pipeline and identified two SNPs were independent (Table 1). One SNP was identified in the combined analysis and the other one SNP in the female-only analysis. The LD patterns analysis showed rs111708875 was only in linkage with four SNPs in female-only analysis (Supplementary Fig. 6A), and the rs111708875 carriers exhibited a higher risk with an HR of 4.14 (95% CI = 2.55 – 6.67) and *P*-value = 5.20×10^−9^(Fig. 3). rs3128076 showed linkage disequilibrium with 33 SNPs, and many of these SNPs also exhibited X-chromosome-wide associations with motor impairment in combined analysis (Supplementary Fig. 6B). It should be noted that the rs3128076 carriers showed a reduced risk of motor impairment (HR = 0.72, 95% CI = 0.62–0.84, *P*-value = 3.19×10^−5^) (Supplementary Fig. 7).

The two independent SNPs were still considered to be associated with HY3 in the PPMI or HBS cohort using CoxPH analyses. We observed that rs3128076 was associated with HY3 in the combined analysis in the HBS cohort, while rs111708875 was associated with HY3 in female-only analysis in the PPMI cohort (*P*-value < 0.05) (Supplementary Table 2).

Among the 485 SNPs identified as genetically associated with the risk of developing PD, AD, or LBD, none overlapped with our two SNPs associated with HY3, nor with the 49 SNPs in high LD with the HY3-associated variants (r^2^ > 0.6).

### Exploratory functional analyses prioritized variants

We utilized eQTL summary statistics data from 49 tissues provided by the GTEx and five studies in the eQTL catalogue (Methods). Of the 40 identified variants (34 from GCI and six from HY3), one was significantly associated with gene expression in GTEx (FDR < 0.05, Supplementary Table 4). The independent SNP rs142724191 in the GCI combined and male-only analyses have been identified as being linked to the regulation of *GSPT2* gene expression (FDR = 0.00013). Colocalization analysis revealed that no posterior probabilities of > 0.70 were identified, indicating a lack of evidence supporting the hypothesis that the identified variants in the eQTL and XWSS datasets were associated with a shared causal variant (Supplementary Table 4).

## Discussion

Our XWSS of 15 longitudinal PD cohorts highlighted the associations between X chromosome variants and cognitive or motor decline during PD progression. Specifically, we revealed distinct genetic risk loci associated with the progression of PD in the stratified and combined analyses. These variants underscored the idea that patients with male vs. female have differences in the genetic architecture of progression.

We discovered 11 independent SNPs associated with cognitive decline in PD patients. Notably, these SNPs did not overlap with previously reported variants associated with susceptibility to PD^25, 26^. Moreover, they did not overlap with variants associated with AD^35^, or LBD^33^. We conducted LD analysis between the risk SNPs (PD, AD, or LBD) and the independent SNPs found in the GCI analysis, and did not observe any overlap between the two sets of SNPs. A similar observation was made in the HY3 analysis, where the identified two SNPs were not found in prior PD, AD, or LBD studies, and no intersection with known PD, AD, or LBD variants was observed in the LD analysis. Thus, our study nominates novel candidate progression loci. Among the 13 independent SNPs, we observed partially consistent associations in the PPMI or HBS cohort, supporting the robustness of our findings. Several SNPs, such as rs142724191, rs144112368 and rs111708875, demonstrated reproducible associations with GCI or HY3 in stratified or combined analyses. Nonetheless, incomplete validation of other SNPs could be attributed to limited sample sizes and cohort heterogeneity, as reflected by instances of convergence failure in the Cox regression analysis. *AC003035.1*, *RP11–104D21.3*, and *RP11–56H2.2* are long non-coding RNA (lncRNA) genes located near the variants rs138116640, rs142724191, and rs185903733, respectively. LncRNAs have been implicated in PD^44, 45^, and further studies of these lncRNAs are needed to elucidate their specific functions and potential associations with the progression of PD.

We uncovered an X-chromosome-wide association variant rs72616437 in the female-only GCI analyses. This rs72616437 variant, located near the *ATP2B3* gene, can mitigate erastin-induced ferroptosis in HT-22 cells^46^. Evidence suggests that ferroptosis plays a critical role in the development of neurological disorders, including PD and AD^47, 48^. This process is mediated via the P62-KEAP1-NRF2-HO-1 pathway, highlighting a potential genetic target for PD research^46^. Another SNP rs111708875 reached a genome-wide significant association with motor impairment in female-only analysis, near *COL4A5* which encodes one of the six subunits of type IV collagen. The mutations in *COL4A5* are linked to X-Linked Alport Syndrome^49^, but its role in the PD has yet to be documented.

For the eQTL analysis, rs142724191 variant was associated with *GSPT2* in nerve tibial tissues. A deletion in the Xp11.22 region, which includes the *GSPT2* gene, has been linked to syndromic X-linked intellectual disability^50–52^. Patients with this syndrome commonly present with intellectual disability, developmental delay, and other neurobehavioral abnormalities. These clinical features suggest that the underlying pathological mechanisms may overlap with those implicated in PD.

There are several limitations in our study. The reports of X chromosome variants associated with motor and cognitive impairment were based on phase I data in the International Genetics of Parkinson Disease Progression Consortium. Studies with large PD longitudinal cohorts are required to confirm and replicate our findings. Another limitation of our study was its focus on common variants, and lack of analysis of the effects of rare variants on the X chromosome due to the limitation of imputation using the genotyping array. Moreover, patients with PD in this exploratory study are predominantly from North American and European cohorts, which may limit the generalizability of our findings across diverse ethnicities and populations. The future availability of genomic data from diverse ancestry of PD longitudinal cohorts, together with advanced technologies for analyzing admixed populations, will enhance the understanding of the X chromosome-linked genetic architecture of the progression of PD.

In summary, our findings complement previously reported autosomal genetic risk loci for the progression of PD. These results suggest that variants on the X chromosome may modulate the PD progression and that they may contribute, in part, to the differences observed in the natural course of female and male PD patients.

## Supplementary Material

Supinfo Supplementary Fig. 1: The pipeline for X Chromosome genotyping data quality control.
Supplementary Fig. 2: QQ plots of X chromosome common variants associated with global cognitive impairment.
Supplementary Fig. 3: LocusZoom plot of rs138116640 in the combined analysis and rs193287960 in the male-only analysis for GCI.
Supplementary Fig. 4: Covariate-adjusted survival curves for 11 independently significant SNPs for GCI.
Supplementary Fig. 5: QQ plots of X chromosome common variants associated with motor impairment.
Supplementary Fig. 6: LocusZoom plot of rs111708875 in the female-only analysis and rs3128076 in the combined analysis for HY3.
Supplementary Fig. 7: Covariate-adjusted survival curves for one independently significant SNPs for HY3.
Supplementary Table 1 Overview of study cohorts.
Supplementary Table 2 Reanalysis of 13 independent progression loci in HBS or PPMI cohort.
Supplementary Table 3 Known X chromosome susceptibility variants associated with PD, AD or LBD.
Supplementary Table 4 The *cis*-eQTLs pairs between significant XWSS SNPs and genes in GTEx.

## Figures and Tables

**Table 1:** Progression loci from combined and stratified XWSS.

SNP	ChrX Pos (hg19)	EA/OA	EAF	Nearest gene	HR combined (95% CI)	*P*-value combined	HR male-only (95% CI)	*P*-value male-only	HR female-only (95% CI)	*P*-value female-only	Endpoint event

rs138116640	14110840	C/T	0.014	*AC003035.1*	2.06(1.54–2.75)	1.15×10^−6^	2.08(1.52–2.84)	4.38×10^−6^	1.74(0.75–4.01)	0.20	GCI
rs142724191	50992700	G/A	0.025	*RP11-104D21.3*	1.73(1.38–2.15)	1.26×10^−6^	1.72(1.35–2.18)	9.01×10^−6^	2.43(1.22–4.84)	0.11	GCI
rs144112368	146251735	T/C	0.020	*MIR513C*	2.18(1.64–2.88)	5.71×10^−8^	2.18(1.60–2.99)	1.02×10^−6^	1.95(1.04–3.67)	0.04	GCI
rs185903733	51305396	G/T	0.012	*RP11-56H2.2*	1.92(1.42–2.60)	1.97×10^−5^	2.06(1.52–2.81)	4.25×10^−6^	1.25(0.39–3.97)	0.71	GCI
rs193287960	14048156	C/G	0.018	*GEMIN8*	1.90(1.44–2.51)	6.32×10^−6^	2.00(1.50–2.67)	2.18×10^−6^	1.10(0.37–3.30)	0.86	GCI
rs145104202	130970471	T/C	0.012	*RP11-453F18__B.1*	1.95(1.42–2.68)	3.51×10^−5^	2.13(1.54–2.95)	4.36×10^−6^	1.04(0.33–3.26)	0.95	GCI
rs142241351	22572294	T/A	0.027	*RP11-40F8.2*	1.17(0.91–1.51)	0.21	0.98(0.71–1.35)	0.90	3.35(1.98–5.66)	6.01×10^−6^	GCI
rs4892830	2934940	A/G	0.028	*ARSH*	1.24(1.00–1.55)	0.05	0.97(0.73–1.30)	0.86	2.62(1.71–4.01)	8.92×10^−6^	GCI
rs72616437	152817031	C/T	0.014	*ATP2B3*	1.06(0.72–1.58)	0.76	0.72(0.41–1.28)	0.26	5.64(2.66–11.97)	6.71×10^−6^	GCI
rs146491961	8253284	A/C	0.014	*VCX2*	1.45(0.99–2.13)	0.06	0.99(0.56–1.76)	0.98	4.52(2.33–8.74)	7.81×10^−6^	GCI
rs778709493	101830586	C/T	0.011	*NXF4*	1.71(1.13–2.59)	0.01	1.05(0.52–2.12)	0.89	4.82(2.45–9.48)	5.40×10^−6^	GCI
rs3128076	34976473	C/T	0.270	*FAM47B*	0.81(0.74–0.89)	6.67×10^−6^	0.81(0.73–0.90)	4.87×10^−5^	0.82(0.69–0.99)	0.04	HY3
rs111708875	107766257	G/A	0.016	*COL4A5*	1.16(0.89–1.51)	0.26	0.78(0.52–1.16)	0.21	3.98(2.54–6.25)	1.84×10^−9^	HY3

*P*-value from the Cox proportional hazards statistic used to estimate the influence of SNP on time (years from onset of PD) to reaching the endpoint of GCI as indicated by a MMSE ≤ 25 and motor impairment as indicated by Hoehn and Yahr stage = 3. For HY3 analysis, we corrected test statistics based on the LDSC intercept. Only independent SNPs were displayed. HR = hazard ratio; EA/OA = effect allele/other Alleles; EAF = Effect allele frequency. The SNP rs142724191 and rs144112368 passed the X-chromosome-wide threshold in both combined and male-only

## Data Availability

The genotype and clinical data for PPMI included in this study are publicly available upon request at ppmi@loni.usc.edu. Clinical data for the Parkinson’s disease biomarker program (PDBP) included in this study are publicly available through https://pdbp.ninds.nih.gov. Clinical longitudinal data and genotyping data for the other cohorts included are accessible through appropriate data-sharing agreements that protect patient privacy with the institutions that conducted or are conducting study consents and clinical assessments under local institutional review board approvals. Colocalization datasets are available from eQTL catalogue (https://www.ebi.ac.uk/eqtl/) and GTEx (https://www.gtexportal.org/home/).
