# Supplementary material for: Genetic analysis of the X chromosome associates loci with progression of Parkinson’s disease
Source: Mov Disord. Author manuscript; Available in PMC 2026 Jun 3. (PMC12353966; doi:10.1002/mds.30252)
Supplement: Supinfo — Supplementary Fig. 1: The pipeline for X Chromosome genotyping data quality control. Supplementary Fig. 2: QQ plots of X chromosome common variants associated with global cognitive impairment. Supplementary Fig. 3: LocusZoom plot of rs138116640 in the combined analysis and rs193287960 in the male-only analysis for GCI. Supplementary Fig. 4: Covariate-adjusted survival curves for 11 independently significant SNPs for GCI. Supplementary Fig. 5: QQ plots of X chromosome common variants associated with motor impairment. Supplementary Fig. 6: LocusZoom plot of rs111708875 in the female-only analysis and rs3128076 in the combined analysis for HY3. Supplementary Fig. 7: Covariate-adjusted survival curves for one independently significant SNPs for HY3. Supplementary Table 1 Overview of study cohorts. Supplementary Table 2 Reanalysis of 13 independent progression loci in HBS or PPMI cohort. Supplementary Table 3 Known X chromosome susceptibility variants associated with PD, AD or LBD. Supplementary Table 4 The cis-eQTLs pairs between significant XWSS SNPs and genes in GTEx. [file NIHMS2082978-supplement-Supinfo.docx]

**Supplementary**

**Genetic analysis of the X chromosome associates loci with progression of Parkinson’s disease**

Yu Liao, BS^1,2^, Hao Wu, MS^1,2^, Junhao Wang, MS^1,2^, Jean-Christophe Corvol, MD^3^, Jodi Maple-Grødem, PhD^4,5^, Meghan C. Campbell, PhD^6^, Alexis Elbaz, MD^7^, Alexis Brice, MD^3^, Michael A. Schwarzschild, MD, PhD^8^, Pille Taba, MD, PhD^9,10^, Sulev Kõks, MD, PhD^11,12^, Thomas G. Beach, MD, PhD^13^, Guido Alves, PhD^4,5,14^, Ole-Bjørn Tysnes, MD^15,16^, Joel S. Perlmutter, MD^6,17,18^, Baijayanta Maiti, PhD^6^, Jacobus J. van Hilten, MD^19^, Roger A. Barker, PhD^20,21^, Caroline H. Williams-Gray, PhD^20^, Clemens R. Scherzer, MD^22,23,24,25^, and Ganqiang Liu, PhD^1,2,26*^ for the International Genetics of Parkinson Disease Progression (IGPP) Consortium.

^1^Shenzhen Key Laboratory of Systems Medicine in Inflammatory Diseases, School of Medicine, Shenzhen Campus of Sun Yat-sen University, Shenzhen, Guangdong, 518107, China

^2^Department of Medical Informatics and Neurobiology Research Center, School of Medicine, Shenzhen Campus of Sun Yat-sen University, Shenzhen, Guangdong, 518107, China

^3^Sorbonne Université, Institut du Cerveau – Paris Brain Institute - ICM, Institut National de la Santé et de la Recherche Médicale, Centre National de la Recherche Scientifique, Assistance Publique Hôpitaux de Paris, Département de Neurologie et de Génétique, Hôpital Pitié-Salpêtrière, F-75013, Paris, France.

^4^The Centre for Movement Disorders, Centre for Brain Health, Stavanger University Hospital, 4011, Stavanger, Norway.

^5^Department of Chemistry, Bioscience and Environmental Engineering, University of Stavanger, 4021, Stavanger, Norway.

^6^Departments of Neurology and Radiology, Washington University School of Medicine, St. Louis, MO 63110, USA.

^7^Université Paris-Saclay, UVSQ, Inserm, Gustave Roussy, CESP, 94805, Villejuif, France.

^8^Department of Neurology, Massachusetts General Hospital and Harvard Medical School, Boston, MA 02114, USA.

^9^Department of Neurology and Neurosurgery, Institute of Clinical Medicine, University of Tartu, Tartu, 50406, Estonia.

^10^Neurology Clinic, Tartu University Hospital, Tartu, 50406, Estonia.

^11^Centre for Molecular Medicine and Innovative Therapeutics, Murdoch University, Murdoch, Perth, 6150 WA, Australia.

^12^Perron Institute for Neurological and Translational Science, Nedlands, WA 6009, Australia.

^13^Banner Sun Health Research Institute, Sun City, AZ, USA.

^14^Department of Neurology, Stavanger University Hospital, 4068, Stavanger, Norway.

^15^Department of Neurology, Haukeland University Hospital, 5020, Bergen, Norway.

^16^Department of Clinical Medicine, University of Bergen, 5020, Norway.

^17^Departments of Radiology and Neuroscience, Washington University School of Medicine, St. Louis, MO 63110, USA.

^18^Program of Physical Therapy and Program of Occupational Therapy, Washington University School of Medicine, St. Louis, MO 63110, USA.

^19^Department of Neurology, Leiden University Medical Center, Albinusdreef 2, 2333 ZA, Leiden, The Netherlands.

^20^John Van Geest Centre for Brain Repair, Department of Clinical Neurosciences, University of Cambridge, Cambridge, CB2 0PY, UK.

^21^Wellcome - MRC Cambridge Stem Cell Institute, University of Cambridge, Cambridge, CB2 0AW, UK.

^22^Stephen & Denise Adams Center for Parkinson’s Disease Research of Yale School of Medicine, New Haven, CT 06510, USA

^23^APDA Center for Parkinson Precision Medicine, Yale, New Haven, CT 06510, USA

^24^Department of Neurology, Yale, New Haven, CT 06510, USA

^25^Department of Genetics, Yale, New Haven, CT 06510, USA

^26^Guangdong Provincial Key Laboratory of Brain Function and Disease, Guangzhou, 510080, China

*Correspondence should be addressed to:

Ganqiang Liu, Ph.D.

School of Medicine, Shenzhen Campus of Sun Yat-sen University

No.66, Gongchang Road, Guangming Distrct

Shenzhen, Guangdong, 518107, China

Phone: 86-0755-23260316

Email: [liugq3@mail.sysu.edu.cn](mailto:liugq3@mail.sysu.edu.cn)

# CONTENTS

[CONTENTS 4](#_Toc196165538)

[Supplementary Method 5](#_Toc196165539)

[Chromosome X genotyping quality control pipeline 5](#_Toc196165540)

[Colocalization analyses 6](#_Toc196165541)

[Acknowledgements 6](#_Toc196165542)

[Supplementary Figures 12](#_Toc196165543)

[Supplementary Fig. 1: The pipeline for X Chromosome genotyping data quality control. 12](#_Toc196165544)

[Supplementary Fig. 2: QQ plots of X chromosome common variants associated with global cognitive impairment. 13](#_Toc196165545)

[Supplementary Fig. 3: LocusZoom plot of rs138116640 in the combined analysis and rs193287960 in the male-only analysis for GCI. 14](#_Toc196165546)

[Supplementary Fig. 4: Covariate-adjusted survival curves for 11 independently significant SNPs for GCI. 15](#_Toc196165547)

[Supplementary Fig. 5: QQ plots of X chromosome common variants associated with motor impairment. 16](#_Toc196165548)

[Supplementary Fig. 6: LocusZoom plot of rs111708875 in the female-only analysis and rs3128076 in the combined analysis for HY3. 17](#_Toc196165549)

[Supplementary Fig. 7: Covariate-adjusted survival curves for one independently significant SNPs for HY3. 18](#_Toc196165550)

[Supplementary Table 19](#_Toc196165551)

[Supplementary Table 1 Overview of study cohorts. 19](#_Toc196165552)

[Supplementary Table 2 Reanalysis of 13 independent progression loci in HBS or PPMI cohort. 20](#_Toc196165553)

[Supplementary Table 3 Known X chromosome susceptibility variants associated with PD, AD or LBD. 21](#_Toc196165554)

[Supplementary Table 4 The *cis*-eQTLs pairs between significant XWSS SNPs and genes in GTEx. 22](#_Toc196165555)

[References 23](#_Toc196165556)

# Supplementary Method

## Chromosome X genotyping quality control pipeline

*Pre-imputation quality control.* We performed a QC prior to X chromosome imputation for 4,020 patients with genotyping array by PLINK. We used 49,326 single nucleotide polymorphisms (SNPs) on the X chromosome and then removed 0 variants in Pseudoautosomal regions (PARs) (<https://github.com/SexChrLab/SexChrCoordinates>). 46 SNPs with Hardy-Weinberg equilibrium (HWE) *P*-value < 10^−6^ in females were excluded across 4,020 PD patients, 183 SNPs with a genotyping rate < 95% were removed, and none individuals with > 10% genotype missingness.

*Imputation*. The 49,097 remaining SNPs in 4,020 patients were imputed by Minimac4 (Phasing by Eagle v2.4) using reference panels HRC r1.1(hg19) with the European subpanel on the Michigan Imputation Server (v1.2.4)^1^. The 798,555 SNPs were remained with imputation score of R^2^ ≥ 0.3.

*ChrX post-imputation quality control.* We carried out QC to filter SNPs after imputation: (1) 25,679 SNPs in completed X-linked regions were excluded, which involved ampliconic regions, X transposed regions (XTRs) and PARs; (2) separated genotypes into 2,548 male and 1,472 female subsets; (3) six SNPs that did not meet HWE criteria in females and had a *P*-value < 10^−6^ were excluded across 4,020 PD patients; (4) 118 variants with < 95% genotyping rate, 0 patient with > 10% genotype missingness and 569,536 SNPs with minor allele frequency (MAF) < 1% were excluded in females; (5) 188 variants with < 95% genotyping rate, 0 patient with > 10% genotype missingness and 569,234 SNPs with MAF < 1% were excluded in males. A total of 4,020 PD patients with 218,593 SNPs passed QC in both males and females.

*Merge PPMI PD patients*. The PPMI consists of 471 PD patients that passed in the prior to X-chromosome QC in Liu et al^2^ with the WGS data. We directly used genomic VCF files (calling against Human Genome reference hg19) and extracted 1,413,980 SNPs on the X chromosome. Then, we merged these SNPs with 218,593 SNPs that passed *ChrX post-imputation QC* and retained 214,818 SNPs in 4,491 PD patients.

*Final ChrX* *quality control.* We further conducted a final QC, similar to *ChrX post-imputation quality control*. (1) separated genotypes into 2,838 male and 1,653 female subsets; (2) 270 SNPs that did not meet HWE criteria in females and had a *P*-value < 10^−6^ were excluded across 4,491 PD patients; (3) 10 variants with < 95% genotyping rate, 0 patient with > 10% genotype missingness and 640 SNPs with MAF < 1% were excluded in females; (4) Four variants with < 95% genotyping rate, 0 patient with >10% genotype missingness and 215 SNPs with MAF < 1% were excluded in males; (5) a total of 213,758 SNPs passed QC in both males and females with total 4,491 patients; (6) 24 patients without clinical records were excluded, resulting in 3,996 PD patients with MEGA genotyping data and 471 PD patients with WGS data (total *N* = 4,467).

## Colocalization analyses

We conducted colocalization analyses to evaluate whether genetic variants associated with GCI or HY3 overlap with regulatory elements across various tissue types. The analyses were conducted for all genes located within a 2-Mb window centered on X-chromosome-wide significant variants for each identified locus. These analyses were performed using the “coloc.abf” function within the “coloc” R package (version 5.1.0.1)^3^. Given the absence of a control group for the XWSS data, beta coefficients and standard errors were utilized. For other datasets, we incorporated sample size, *P*-value, and minor allele frequency (MAF) as input parameters.

Colocalization was deemed to have significant evidence if the colocalization posterior probability (PP4) > 0.70 for each dataset and tissue type. Specifically, significance in the GTEx was determined using false discovery rate (FDR) correction, whereas a *P*-value threshold of < 10^-5^ was employed for five datasets from the eQTL catalogue. This threshold was chosen to approximate the significance level of FDR-corrected QTL *P*-values, given that direct FDR-corrected *P*-values were not provided.

## Acknowledgements

Harvard Biomarkers Study. Co-Directors: Brigham and Women’s Hospital: Clemens R. Scherzer, Massachusetts General Hospital: Bradley T. Hyman; Investigators and Study Coordinators: Brigham and Women’s Hospital: Yuliya Kuras, Karbi Choudhury, Nada Laroussi, Daly Franco, Michael T. Hayes, Nutan Sharma, Vikram Khurana, Claudio Melo De Gusmao, Chizoba C. Umeh, Reisa Sperling; Massachusetts General Hospital: John H. Growdon, Michael A. Schwarzschild, Albert Y. Hung, Aleksandar Videnovic, Alice W. Flaherty, Deborah Blacker, Anne-Marie Wills, Steven E. Arnold, Ann L. Hunt, Nicte I. Mejia, Anand Viswanathan, Stephen N. Gomperts, Mark W. Albers, Maria Allora-Palli, David Hsu, Alexandra Kimball, Scott McGinnis, John Becker, Randy Buckner, Thomas Byrne, Maura Copeland, Bradford Dickerson, Matthew Frosch, Theresa Gomez-Isla, Steven Greenberg, Julius Hedden, Elizabeth Hedley-Whyte, Keith Johnson, Raymond Kelleher, Aaron Koenig, Maria Marquis-Sayagues, Gad Marshall, Sergi Martinez-Ramirez, Donald McLaren, Olivia Okereke, Elena Ratti, Christopher William, Koene Van Dij, Shuko Takeda, Anat Stemmer-Rachaminov, Jessica Kloppenburg, Catherine Munro, Rachel Schmid, Sarah Wigman, Sara Wlodarcsyk; Data Coordination: Brigham and Women’s Hospital: Thomas Yi; Biobank Management Staff: Brigham and Women’s Hospital: Idil Tuncali. We thank all study participants and their families for their invaluable contributions. HBS is made possible by generous support from the Harvard NeuroDiscovery Center, with additional contributions from the Michael J Fox Foundation, NINDS U01NS082157, U01NS100603, and the Massachusetts Alzheimer’s Disease Research Center NIA P50AG005134.

DIGPD: Steering committee: Jean-Christophe Corvol (Pitié-Salpêtrière Hospital, Paris, principal investigator of DIGPD), Alexis Elbaz (CESP, Villejuif, member of the steering committee), Marie Vidailhet (Pitié-Salpêtrière Hospital, Paris, member of the steering committee), Alexis Brice (Pitié-Salpêtrière Hospital, Paris, member of the steering committee and PI for genetic analysis) ; Statistical analyses: Alexis Elbaz (CESP, Villejuif, PI for statistical analyses), Fanny Artaud (CESP, Villejuif, statistician); Principal investigators for sites (alphabetical order): Frédéric Bourdain (CH Foch, Suresnes, PI for site), Jean-Philippe Brandel (Fondation Rothschild, Paris, PI for site), Jean-Christophe Corvol (Pitié-Salpêtrière Hospital, Paris, PI for site), Pascal Derkinderen (CHU Nantes, PI for site), Franck Durif (CHU Clermont-Ferrand, PI for site), Richard Levy (CHU Saint-Antoine, Paris, PI for site), Fernando Pico (CH Versailles, PI for site), Olivier Rascol (CHU Toulouse, PI for site); Co-investigators (alphabtical order): Anne-Marie Bonnet (Pitié-Salpêtrière Hospital, Paris, site investigator), Cecilia Bonnet (Pitié-Salpêtrière Hospital, Paris, site investigator), Christine Brefel-Courbon (CHU Toulouse, site investigator), Florence Cormier-Dequaire (Pitié-Salpêtrière Hospital, Paris, site investigator), Bertrand Degos (Pitié-Salpêtrière Hospital, site investigator), Bérangère Debilly (CHU Clermont-Ferrand, site investigator), Alexis Elbaz (Pitié-Salpêtrière Hospital, Paris, site investigator), Monique Galitsky (CHU de Toulouse, site investigator), David Grabli (Pitié-Salpêtrière Hospital, Paris, site investigator), Andreas Hartmann (Pitié-Salpêtrière Hospital, Paris, site investigator), Stephan Klebe (Pitié-Salpêtrière Hospital, Paris, site investigator), Julia Kraemmer (Pitié-Salpêtrière Hospital, site investigator), Lucette Lacomblez (Pitié-Salpêtrière Hospital, Paris, site investigator), Sara Leder (Pitié-Salpêtrière Hospital, Paris, site investigator), Graziella Mangone (Pitié-Salpêtrière Hospital, Paris, site investigator), Louise-Laure Mariani (Pitié-Salpêtrière Hospital, Paris, site investigator), Ana-Raquel Marques (CHU Clermont Ferrand, site investigator), Valérie Mesnage (CHU Saint Antoine, Paris, site investigator), Julia Muellner (Pitié-Salpêtrière Hospital, Paris, site investigator), Fabienne Ory-Magne (CHU Toulouse, site investigator), Violaine Planté-Bordeneuve (Henri Mondor Hospital, Créteil, site investigator), Emmanuel Roze (Pitié-Salpêtrière Hospital, Paris, site investigator), Melissa Tir (CH Versailles, site investigator), Marie Vidailhet (Pitié-Salpêtrière Hospital, Paris, site investigator), Hana You (Pitié-Salpêtrière Hospital, Paris, site investigator); Neuropsychologists: Eve Benchetrit (Pitié-Salpêtrière Hospital, Paris, neuropsychologist), Julie Socha (Pitié-Salpêtrière Hospital, Paris, neuropsychologist), Fanny Pineau (Pitié-Salpêtrière Hospital, Paris, neuropsychologist), Tiphaine Vidal (CHU Clermont-Ferrand, neuropsychologist), Elsa Pomies (CHU de Toulouse, neuropsychologist), Virginie Bayet (CHU de Toulouse, neuropsychologist); Genetic core: Alexis Brice (Pitié-Salpêtrière Hospital, Paris, PI for genetic studies), Suzanne Lesage (INSERM, ICM, Paris, genetic analyses), Khadija Tahiri (INSERM, ICM, Paris, lab technician) Hélène Bertrand (INSERM, ICM, Paris, lab technician), Graziella Mangone (Pitié-Salpêtrière Hospital, Paris, genetic analyses); Sponsor activities and clinical research assistants: Alain Mallet (Pitié-Salpêtrière Hospital, Paris, sponsor representative), Coralie Villeret (Hôpital Saint Louis, Paris, Project manager), Merry Mazmanian (Pitié-Salpêtrière Hospital, Paris, project manager), Hakima Manseur (Pitié-Salpêtrière Hospital, Paris, clinical research assistant), Mostafa Hajji (Pitié-Salpêtrière Hospital, Paris, data manager), Benjamin Le Toullec (Pitié-Salpêtrière Hospital, Paris, clinical research assistant), Vanessa Brochard (Pitié-Salpêtrière Hospital, Paris, project manager), Monica Roy (CHU de Nantes, clinical researh assistant), Isabelle Rieu (CHU Clermont-Ferrand, clinical research assistant), Stéphane Bernard (CHU Clermont-Ferrand, clinical research assistant), Antoine Faurie-Grepon (CHU de Toulouse, clnical research assistant). The study was sponsored by the Assistance Publique Hôpitaux de Paris, and was funded by a grant from the Ministry of Health (PHRC AOR0810).

PreCEPT/PostCEPT Study: PreCEPT/PostCEPT Steering Committee: University of Rochester: David Oakes, Ira Shoulson; University of Toronto: Anthony E. Lang; Parlinson’s Institute: Caroline Tanner; Institute for Neurodegenerative Disorders: Kenneth Marek; Voyager Therapeutics: Bernard Ravina; Brigham and Women’s Hospital: Clemens Scherzer, University of Ottawa: Michael Schlossmacher, Avid Radiopharmaceuticals: Andrew Siderowf, We thank the Parkinson Study Group (PSG) PreCEPT/PostCEPT investigators for the acquisition of high-quality clinical data, careful follow up of study subjects and collection of blood samples.

DATATOP: We thank the investigators of the Parkinson Study Group (PSG) DATATOP for the acquisition of high-quality clinical data, careful follow-up of study subjects, and DNA collection in the DATATOP cohort.

PICNICS: Investigators: Roger Barker, Caroline Williams-Gray, David P Breen, Gemma Cummins, Jonathan Evans, Sophie Winder-Rhodes, Ruwani Wijeyekoon, Marta Camacho. The PICNICS study was sponsored by the University of Cambridge/Cambridge University Hospitals NHS Trust UK and received funding from the Cure Parkinson's Trust, the Van Geest Foundation, Parkinson's UK, and the NIHR Cambridge Biomedical Research Centre (NIHR203312). The views expressed are those of the authors and not necessarily those of the NHS, the NIHR or the Department of Health.

CamPaIGN: Investigators: Roger Barker, Tom Foltynie, Caroline Williams-Gray, Trevor Robbins, Carol Brayne, Sarah Mason, Sophie Winder-Rhodes, Ruwani Wijeyekoon. The CamPaIGN study was sponsored by the University of Cambridge/Cambridge University Hospitals NHS Trust UK and has received funding from the Wellcome Trust, the Medical Research Council, the Patrick Berthoud Trust, and the NIHR Cambridge Biomedical Research Centre (NIHR203312). The views expressed are those of the authors and not necessarily those of the NHS, the NIHR or the Department of Health.

PROPARK/PROPARK-C: The PROPARK study was headed by Jacobus J. van Hilten and Johan Marinus.

PDBP: Data and biospecimens used in preparation of this manuscript were obtained from the Parkinson's Disease Biomarkers Program (PDBP) Consortium, supported by the National Institute of Neurological Disorders and Stroke at the National Institutes of Health. Investigators include: Roger Albin, Roy Alcalay, Alberto Ascherio, Thomas Beach, Sarah Berman, Bradley Boeve, F. DuBois Bowman, Shu Chen, Alice Chen-Plotkin, William Dauer, Ted Dawson, Paula Desplats, Richard Dewey, Ray Dorsey, Jori Fleisher, Kirk Frey, Douglas Galasko, James Galvin, Dwight German, Steven Gunzler, Lawrence Honig, Xuemei Huang, David Irwin, Kejal Kantarci, Anumantha Kanthasamy, Daniel Kaufer, Qingzhong Kong, James Leverenz, Allan Levey, Carol Lippa, Irene Litvan, Oscar Lopez, Jian Ma, Lara Mangravite, Karen Marder, Nandakumar Narayanan, Laurie Orzelius, Vladislav Petyuk, Judith Potashkin, Liana Rosenthal, Rachel Saunders-Pullman, Clemens Scherzer, Michael Schwarzschild, Nicholas Seyfried, Tanya Simuni, Andrew Singleton, David Standaert, Debby Tsuang, David Vaillancourt, Jerrold Vitek, David Walt, Andrew West, Cyrus Zabetian, and Jing Zhang. The PDBP Investigators have not participated in reviewing the data analysis or content of the manuscript.

PPMI: Data used in the preparation of this article were obtained [06/16/2017] from the Parkinson's Progression Markers Initiative (PPMI) database (www.ppmi-info.org/access-data- specimens/download-data), RRID: SCR_006431. For up-to-date information on the study, visit www.ppmi-info.org. PPMI – a public-private partnership – is funded by the Michael J. Fox Foundation for Parkinson's Research and funding partners, including 4D Pharma, Abbvie, AcureX, Allergan, Amathus Therapeutics, Aligning Science Across Parkinson's, AskBio, Avid Radiopharmaceuticals, BIAL, Biogen, Biohaven, BioLegend, BlueRock Therapeutics, Bristol-Myers Squibb, Calico Labs, Celgene, Cerevel Therapeutics, Coave Therapeutics, DaCapo Brainscience, Denali, Edmond J. Safra Foundation, Eli Lilly, Gain Therapeutics, GE HealthCare, Genentech, GSK, Golub Capital, Handl Therapeutics, Insitro, Janssen Neuroscience, Lundbeck, Merck, Meso Scale Discovery, Mission Therapeutics, Neurocrine Biosciences, Pfizer, Piramal, Prevail Therapeutics, Roche, Sanofi, Servier, Sun Pharma Advanced Research Company, Takeda, Teva, UCB, Vanqua Bio, Verily, Voyager Therapeutics, the Weston Family Foundation and Yumanity Therapeutics.

Arizona Study of Aging/Brain and Body Donation Program: National Institute of Neurological Disorders and Stroke, U24 NS072026 National Brain and Tissue Resource for Parkinson’s Disease and Related Disorders; National Institute on Aging, P30 AG19610 Arizona Alzheimer’s Disease Core Center; Arizona Department of Health Services, Arizona Alzheimer’s Consortium; Arizona Biomedical Research Commission, Arizona Parkinson's Disease Consortium; Michael J. Fox Foundation for Parkinson’s Research.

NET-PD LS1: We would like to thank the patients and families who participated in the NET-PD LS1 study. The following additional NINDS grants supported the Net-PD LS-1 study: U01NS043127, U01NS043128, and U10NS44415-44555 from the National Institute of Neurologic Disorders and Stroke.

Tartu/Perron: We would like to thank all patients and families who participated in the study. Support by the following grants and funders: institutional research grants PRG957 and IUT20–46 of the Estonian Research Council, H2020 ERA-chair grant (agreement 668989, project Transgeno), MSWA, The Michael J. Fox Foundation, Shake It Up Australia and The Perron Institute.

ParkWest: Principal investigators: Guido Alves (Norwegian Centre for Movement Disorders, Stavanger University Hospital), Ole-Bjørn Tysnes (Haukeland University Hospital). Investigators and study coordinators: Karen Herlofson, Solgunn Ongre, Siri Bruun (Sørlandet Hospital Arendal); Ineke HogenEsch, Marianne Kjerandsen, Liv Kari Håland (Haugesund Hospital); Wenche Telstad, Aliaksei Labusau, Jane Kastet (Førde Hospital); Bernd Müller, Geir Olve Skeie, Charalampos Tzoulis (Haukeland University Hospital); Kenn Freddy Pedersen, Michaela Dreetz Gjerstad, Elin Bjelland Forsaa, Jodi Maple-Grødem, Johannes Lange, Veslemøy Hamre Frantzen, Anita Laugaland, Karen Simonsen, Ingvild Dalen (Stavanger University Hospital). The ParkWest study has received funding from the Western Norway Regional Health Authority (grant number 911218), and the Norwegian Parkinson's Disease Association, and the Research Council of Norway (grant number 287842).

PIB funding: NIH grants: NS075321 and NS097437, the American Parkinson Disease Association (APDA), the Greater St. Louis Chapter of the APDA, the Barnes Jewish Hospital Foundation (Elliot Stein Family Fund, Parkinson disease research fund).

# Supplementary Figures

## Supplementary Fig. 1: The pipeline for X Chromosome genotyping data quality control.

We conducted quality control on both PD patients and SNPs, encompassing five major procedures: pre-imputation quality control, imputation, chrX post-imputation quality control, merged WGS data of PD patients from the PPMI cohort with genotyping data, and then conducted final ChrX quality control. *The details of prior to X-chromosome quality control were in ref^2^ Extended Data Fig.1. In total, 4,467 PD cases with 213,758 SNPs on chromosome X passed data quality control.

## Supplementary Fig. 2: QQ plots of X chromosome common variants associated with global cognitive impairment.

QQ plots of X chromosome common variants associated with global cognitive impairment (GCI, MMSE≤25) in the combined (A), male-only (B), and female-only analysis(C) by X-chromosome wide survival study (XWSS). The x-axis represents the expected -log_10_(*P*-value), while the y-axis represents the actual observed -log_10_(*P*-value). The genomic inflation factor (λ_GC_) was calculated using the median chi-squared statistic from the association test results, as implemented in the “qq” function of the “qqman” package in R (version 0.1.8). A lambda value close to 1 indicates little to no inflation of type I error rates, suggesting that population stratification and other confounding factors are well controlled. Genomic inflation was quantified through the linkage disequilibrium score regression (LDSC) intercept. The LDSC intercepts for the combined (1.0003, standard error (SE) = 0.032), male-only (0.9773, SE = 0.028) and female-only analysis (1.0027, SE = 0.033) were lower than the λ_GC_, supporting polygenicity as the primary contributor to the observed test statistic inflation.

## Supplementary Fig. 3: LocusZoom plot of rs138116640 in the combined analysis and rs193287960 in the male-only analysis for GCI.

LocusZoom plot of rs138116640 in the combined analysis (A) and rs193287960 in the male-only analysis (B). The x-axis represents the positions of SNPs (hg19), while the y-axis represents the -log_10_(*P*-value) calculated by XWSS. Purple dots represent independent SNPs, while the colors of the remaining dots represent the degree of correlation with each respective point. The hg19/1000 genomes EUR.v3 were used as LD population on LocusZoom plots.

## Supplementary Fig. 4: Covariate-adjusted survival curves for 11 independently significant SNPs for GCI.

Covariate-adjusted survival curves of rs138116640 in the combined analysis (A) and rs193287960 in the male-only analysis (B) for GCI. Other nine SNPs including rs142724191 and rs144112368 in the combined analysis(C-D), rs142724191, rs185903733, rs145104202 and rs144112368 in the male-only analysis(E-H), rs4892830, rs146491961, rs142241351, rs778709493 and rs72616437 in the female-only analysis(I-M). The x-axis represents the years since disease onset, while the y-axis represents the percentage of patients surviving free of global cognitive impairment (MMSE score ≤ 25).

## Supplementary Fig. 5: QQ plots of X chromosome common variants associated with motor impairment.

QQ plots of X chromosome common variants associated with motor impairment (Hoehn and Yahr stage = 3) in the combined (A), male-only (C), and female-only analysis(E) by X-chromosome wide survival study (XWSS). The corresponding genomic inflation factors (λ_GC_) corrected by linkage disequilibrium score regression (LDSC) intercepts are displayed in panels B, D, and F. The x-axis represents the expected -log_10_(*P*-value), while the y-axis represents the actual observed -log_10_(*P*-value). The λ_GC_ was calculated using the median chi-squared statistic from the association test results, as implemented in the “qq” function of the “qqman” package in R (version 0.1.8). A lambda value close to 1 indicates little to no inflation of type I error rates, suggesting that population stratification and other confounding factors are well controlled. Prior to the correction, the LDSC intercepts for the combined (1.111, standard error (SE) = 0.033), male-only (1.089, SE = 0.029) and female-only analysis (1.096, SE = 0.046) exceeded 1.05, indicating potential confounding biases. After correction, the λ_GC_ approached 1.0 (combined: λ_GC_ =1.041; male: λ_GC_ =1.075; female: λ_GC_ =1.009) in all analyses.

## Supplementary Fig. 6: LocusZoom plot of rs111708875 in the female-only analysis and rs3128076 in the combined analysis for HY3.

LocusZoom plot of rs111708875 in the female-only analysis(A) and rs3128076 in the combined analysis(B). The x-axis represents the positions of SNPs (hg19), while the y-axis represents the -log_10_(*P*-value) calculated by XWSS. Purple dots represent independent SNPs, while the colors of the remaining dots represent the degree of correlation with each respective point. The hg19/1000 genomes EUR.v3 were used as LD population on LocusZoom plots.

## Supplementary Fig. 7: Covariate-adjusted survival curves for one independently significant SNPs for HY3.

Covariate-adjusted survival curves of rs3128076 in the combined analysis for motor impairment (Hoehn and Yahr stage = 3). The x-axis represents the year since the onset of Parkinson's disease, while the y-axis represents the percentage of patients surviving free of motor impairment (HY = 3).

# Supplementary Table

## Supplementary Table 1 Overview of study cohorts.

| **Study(Country)** | ***N* (% male)** | | **Age at onset**  **(mean years,**  **SD)** | **Age at**  **Enrollment**  **(mean years, SD)** | **Years of**  **Education**  **(mean years,**  **SD)** | **Study Years**  **(mean years,**  **range)** | **Left-censored Number in**  **GCI analysis** | **Left-censored Number in**  **HY3 analysis** |
| --- | --- | --- | --- | --- | --- | --- | --- | --- |
| HBS (USA) | | 573 (65.3%) | 62.3 (10.5) | 66.2 (10.0) | 15.2 (1.7) | 2.5 (0-10.1) | 29 | 56 |
| NET-PD Long term Study-1 (LS1) (USA, Canada) | | 427 (66.0%) | 58.7 (9.8) | 61.9 (9.6) | 16.1 (3.1) | 4.4 (0-6.3) | 25 | 13 |
| DIGPD (France) | | 376 (60.1%) | 59.9 (9.8) | 62.4 (9.7) | 14.6 (4.7) | 3.3 (0-5.6) | 30 | 6 |
| PROPARK (Netherlands) | | 314 (65.0%) | 53.0 (10.6) | 59.7 (10.7) | 12.0 (4.1) | 4.6 (0-6.3) | 55 | 110 |
| CamPaIGN (UK) | | 119 (54.6%) | 70.0 (9.7) | 70.3 (9.7) | 11.3 (3.3) | 6.4 (0-12.8) | 15 | 16 |
| PICNICS (UK) | | 239 (62.3%) | 68.5 (9.1) | 68.9 (9.2) | 12.2 (3.1) | 3.5 (0-9.0) | 9 | 35 |
| PDBP (USA) | | 301 (62.5%) | 60.2 (9.8) | 65.5 (8.9) | 16.0 (2.6) | 2.4 (0-4.1) | 10 | 24 |
| BannerHealth (USA) | | 147 (66.7%) | 66.1 (10.6) | 76.4 (7.4) | 15.2 (2.6) | 4.2 (0-19.9) | 12 | 21 |
| ParkWest (Norway) | | 165 (64.2%) | 64.7 (9.2) | 67.1 (9.1) | 11.2 (3.2) | 5.0 (3.2-5.6) | 23 | 13 |
| PIB (USA) | | 163 (65.6%) | 62.5 (8.1) | 67.4 (8.1) | 15.6 (2.7) | 2.1 (0-8.1) | 17 | 23 |
| PROPARK-C^&^ (Netherlands) | | 253 (67.6%) | 55.9 (9.8) | 64.3 (8.4) | 11.7 (3.6) | 0 | 4 | 27 |
| DATATOP (USA, Canada) | | 410 (67.6%) | 59.1 (9.2) | 60.2 (8.9) | 14.3 (3.4) | 6.3 (0.1-7.8) | 10 | 0 |
| PreCEPT (USA, Canada) | | 310 (66.5%) | 60.0 (9.3) | 60.8 (9.3) | 16.0 (3.1) | 6.7 (2.5-8.6) | 2 | 0 |
| PPMI (USA) | | 471 (61.6%) | 60.9 (9.8) | 61.8 (9.7) | 15.3 (3.4) | 4.0 (0-6.3) | 11 | 11 |
| Tartu (Estonia)^&^ | | 199 (39.7%) | 66.2 (9.7) | 72.9 (8.2) | 11.0 (5.0) | 0 | 47 | 84 |

The studies included are the Harvard Biomarkers Study (HBS)^4, 5^; Neuroprotection Exploratory Trials in PD- Long term Study-1 (NET-PD LS1)^6^; Drug Interaction with Genes in PD (DIGPD)^7^; PROfiling PARKinson’s disease (PROPARK) study^8^; Cambridgeshire Parkinson’s Incidence from GP to Neurologist (CamPaIGN)^9-11^; Parkinsonism: Incidence, Cognition and Non-motor heterogeneity in Cambridgeshire (PICNICS)^12^; Parkinson’s Disease Biomarkers Program (PDBP)^13^; Banner Health study(Arizona Study of Aging/Brain and Body Donation Program)^14^; ParkWest^15^ and PIB^16^; Deprenyl and Tocopherol Antioxidative Therapy of Parkinsonism (DATATOP)^17^; Parkinson Research Examination of CEP-1347 Trial/A Longitudinal Follow-up of the PRECEPT Study Cohort (PreCEPT/PostCEPT)^18^; Parkinson’s Progression Markers Initiative (PPMI)^19^ and Tartu^20^. ^&^Baseline visit only.

## Supplementary Table 2 Reanalysis of 13 independent progression loci in HBS or PPMI cohort.

| **SNP** | **Analysis** | **HBS**  **HR** | **HBS**  ***P*-value** | **PPMI**  **HR** | **PPMI**  ***P*-value** | **15 cohorts**  **HR** | **15 cohorts**  ***P*-value** | **Endpoint**  **event** |
| --- | --- | --- | --- | --- | --- | --- | --- | --- |
| rs138116640 | combined | 1.69(0.71-4.03) | 0.24 | Inf | 0.99 | 2.06(1.54-2.75) | 1.15×10^-6^ | GCI |
| rs142724191 | combined | 2.67(0.90-7.94) | 0.08 | 1.66(0.78-3.52) | 0.19 | 1.73(1.38-2.15) | 1.26×10^-6^ | GCI |
| rs144112368 | combined | Inf | 0.99 | 3.12(1.21-8.00) | 0.02 | 2.18(1.64-2.88) | 5.71×10^-8^ | GCI |
| rs142724191 | male | 4.44(1.79-11.02) | 0.001 | 1.50(0.67-3.39) | 0.32 | 1.72(1.35-2.18) | 9.01×10^-6^ | GCI |
| rs144112368 | male | Inf | 0.99 | 4.06(1.26-13.10) | 0.02 | 2.18(1.60-2.99) | 1.02×10^-6^ | GCI |
| rs185903733 | male | Inf | 0.99 | 2.80(1.22-6.45) | 0.02 | 2.06(1.52-2.81) | 4.25×10^-6^ | GCI |
| rs193287960 | male | 1.51(0.59-3.87) | 0.39 | Inf | 0.99 | 2.00(1.50-2.67) | 2.18×10^-6^ | GCI |
| rs145104202 | male | Inf | 0.99 | 1.12(0.30-4.17) | 0.87 | 2.13(1.54-2.95) | 4.36×10^-6^ | GCI |
| rs142241351 | female | NA | NA | 2.56(0.30-21.87) | 0.39 | 3.35(1.98-5.66) | 6.01×10^-6^ | GCI |
| rs4892830 | female | NA | NA | 1.80(0.17-19.50) | 0.63 | 2.62(1.71-4.01) | 8.92×10^-6^ | GCI |
| rs72616437 | female | NA | NA | Inf | 0.99 | 5.64(2.66-11.97) | 6.71×10^-6^ | GCI |
| rs146491961 | female | NA | NA | 9.78(0.62-154.82) | 0.11 | 4.52(2.33-8.74) | 7.81×10^-6^ | GCI |
| rs778709493 | female | NA | NA | 2.02(0.10-41.20) | 0.65 | 4.82(2.45-9.48) | 5.40×10^-6^ | GCI |
| rs3128076 | combined | 0.63(0.41-0.96) | 0.03 | 0.85(0.63-1.14) | 0.27 | 0.81(0.74-0.89) | 6.67×10^-6^ | HY3 |
| rs111708875 | female | 3.29(0.32-33.49) | 0.31 | 3.88(1.39-10.83) | 0.01 | 3.98(2.54-6.25) | 1.84×10^-9^ | HY3 |

The 13 independent loci (13 unique SNPs) were identified in the XWSS analysis across all cohorts. We selected the two largest cohorts (PPMI and HBS) in 15 cohorts, and performed Cox proportional hazards analysis of these SNPs in both cohorts, respectively. The "cohort" random effect was excluded, and kept other parameters in the XWSS analysis. NA indicates that the number of events (defined as occurrences of GCI or HY3) in the cohort did not exceed 10, and the hazard ratio (HR) and *P*-value could not be correctly estimated.

## Supplementary Table 3 Known X chromosome susceptibility variants associated with PD, AD or LBD.

| **SNP** | **ChrX Pos (hg19)** | **MAF** | **OR (95% CI)** | ***P*-value** | **Disease** |
| --- | --- | --- | --- | --- | --- |
| rs7066890 | 13892582 | 0.155 | 1.10(1.06–1.13) | 1.20 × 10^−8^ | PD^21^ |
| rs28602900 | 153633533 | 0.115 | 1.10(1.07–1.14) | 1.20 × 10^-9^ | PD^21^ |
| rs525496 | 103276496 | 0.116 | 0.60(0.48–0.77) | 3.13 × 10^-5^ | PD^22^ |
| rs150798997 | 5651167 | 0.003 | 0.64(0.54-0.77) | 2.08 × 10^-6^ | AD^23^ |
| rs12852495 | 10426904 | 0.026 | 1.54(1.28-1.86) | 6.60 × 10^-6^ | AD^23^ |
| rs2142791 | 46550562 | 0.461 | 1.05 (1.04-1.08) | 3.78 × 10^-8^ | AD^23^ |
| rs209215 | 129385813 | 0.399 | 1.05(1.03-1.07) | 2.70 × 10^-6^ | AD^23^ |
| rs5975709 | 135338312 | 0.433 | 0.95(0.94-0.97) | 1.02 × 10^-6^ | AD^23^ |
| rs5930938 | 135462684 | 0.326 | 0.94(0.92-0.97) | 7.55 × 10^-7^ | AD^23^ |
| rs146964414 | 149776634 | 0.082 | 1.10(1.06-1.13) | 8.10 × 10^-8^ | AD^23^ |
| rs141773145 | 19531967 | 0.022 | 2.42(1.65–3.56) | 7.00 × 10^-6^ | LBD^24^ |

The 11 SNPs have been linked to the risk of developing PD, AD or LBD. PD: Parkinson’s Disease; AD: Alzheimer’s Disease (AD); LBD: Lewy Body Dementia.

## Supplementary Table 4 The *cis*-eQTLs pairs between significant XWSS SNPs and genes in GTEx.

| **SNP** | **Type** | **Gene symbol** | **MAF** | **FDR** | **Tissue** | **PP4** |
| --- | --- | --- | --- | --- | --- | --- |
| rs142724191 | Independent SNP in  GCI combined analysis | *GSPT2* | 0.025 | 0.00013 | nerve tibial | 0.0099 |
| rs142724191 | Independent SNP in  GCI male-only analysis | *GSPT2* | 0.025 | 0.00013 | nerve tibial | 1.34×10^-3^ |

For 40 SNPs with X-chromosome association with GCI or HY3 (*P*-value < 9.27 × 10^-6^), we identified one regulated gene in the *cis*-eQTL results of 49 tissues of GTEx. The rs142724191 was the independent SNP after FUMA analysis. FDR: False Discovery Rate corrected *P*-values were directly from GTEx; Tissue: the tissue type where the eQTL was analyzed; PP4: the posterior probability that the same SNP is responsible for both the progression of PD and the gene expression variation. A high PP4 value (close to 1) suggests strong evidence that the SNP is colocalized with the expression of the gene, meaning it may influence both the phenotype and the gene expression.

**References**

1. Das S, Forer L, Schonherr S, et al. Next-generation genotype imputation service and methods. Nat Genet 2016;48:1284-1287.

2. Liu G, Peng J, Liao Z, et al. Genome-wide survival study identifies a novel synaptic locus and polygenic score for cognitive progression in Parkinson's disease. Nat Genet 2021;53:787-793.

3. Giambartolomei C, Vukcevic D, Schadt EE, et al. Bayesian test for colocalisation between pairs of genetic association studies using summary statistics. PLoS Genet 2014;10:e1004383.

4. Locascio JJ, Eberly S, Liao Z, et al. Association between alpha-synuclein blood transcripts and early, neuroimaging-supported Parkinson's disease. Brain 2015;138:2659-2671.

5. Pankratz N, Beecham GW, DeStefano AL, et al. Meta-analysis of Parkinson's disease: identification of a novel locus, RIT2. Ann Neurol 2012;71:370-384.

6. Writing Group for the NETiPDI, Kieburtz K, Tilley BC, et al. Effect of creatine monohydrate on clinical progression in patients with Parkinson disease: a randomized clinical trial. JAMA 2015;313:584-593.

7. Corvol JC, Artaud F, Cormier-Dequaire F, et al. Longitudinal analysis of impulse control disorders in Parkinson disease. Neurology 2018;91:e189-e201.

8. Verbaan D, Marinus J, Visser M, van Rooden SM, Stiggelbout AM, van Hilten JJ. Patient-reported autonomic symptoms in Parkinson disease. Neurology 2007;69:333-341.

9. Williams-Gray CH, Evans JR, Goris A, et al. The distinct cognitive syndromes of Parkinson's disease: 5 year follow-up of the CamPaIGN cohort. Brain 2009;132:2958-2969.

10. Williams-Gray CH, Mason SL, Evans JR, et al. The CamPaIGN study of Parkinson's disease: 10-year outlook in an incident population-based cohort. J Neurol Neurosurg Psychiatry 2013;84:1258-1264.

11. Winder-Rhodes SE, Evans JR, Ban M, et al. Glucocerebrosidase mutations influence the natural history of Parkinson's disease in a community-based incident cohort. Brain 2013;136:392-399.

12. Breen DP, Evans JR, Farrell K, Brayne C, Barker RA. Determinants of delayed diagnosis in Parkinson's disease. J Neurol 2013;260:1978-1981.

13. Rosenthal LS, Drake D, Alcalay RN, et al. The NINDS Parkinson's disease biomarkers program. Mov Disord 2016;31:915-923.

14. Beach TG, Adler CH, Sue LI, et al. Arizona Study of Aging and Neurodegenerative Disorders and Brain and Body Donation Program. Neuropathology 2015;35:354-389.

15. Alves G, Muller B, Herlofson K, et al. Incidence of Parkinson's disease in Norway: the Norwegian ParkWest study. J Neurol Neurosurg Psychiatry 2009;80:851-857.

16. Lucero C, Campbell MC, Flores H, Maiti B, Perlmutter JS, Foster ER. Cognitive reserve and beta-amyloid pathology in Parkinson disease. Parkinsonism Relat Disord 2015;21:899-904.

17. Jankovic J, McDermott M, Carter J, et al. Variable expression of Parkinson's disease: a base-line analysis of the DATATOP cohort. The Parkinson Study Group. Neurology 1990;40:1529-1534.

18. Ravina B, Tanner C, Dieuliis D, et al. A longitudinal program for biomarker development in Parkinson's disease: a feasibility study. Mov Disord 2009;24:2081-2090.

19. Parkinson Progression Marker I. The Parkinson Progression Marker Initiative (PPMI). Prog Neurobiol 2011;95:629-635.

20. Kadastik-Eerme L, Taba N, Asser T, Taba P. The increasing prevalence of Parkinson's disease in Estonia. Acta Neurol Scand 2018;138:251-258.

21. Le Guen Y, Napolioni V, Belloy ME, et al. Common X-Chromosome Variants Are Associated with Parkinson Disease Risk. Ann Neurol 2021;90:22-34.

22. Leal TP, Rao SC, French-Kwawu JN, et al. X-Chromosome Association Study in Latin American Cohorts Identifies New Loci in Parkinson's Disease. Mov Disord 2023;38:1625-1635.

23. Belloy ME, Le Guen Y, Stewart I, et al. Role of the X Chromosome in Alzheimer Disease Genetics. JAMA Neurol 2024;81:1032-1042.

24. Bayram E, Reho P, Litvan I, et al. Genetic analysis of the X chromosome in people with Lewy body dementia nominates new risk loci. NPJ Parkinsons Dis 2024;10:39.
